# Examining the evidence base for burnout

**DOI:** 10.2471/BLT.23.289996

**Published:** 2023-10-04

**Authors:** Renzo Bianchi, Irvin Sam Schonfeld

**Affiliations:** a Department of Psychology, Norwegian University of Science and Technology (NTNU), Dragvoll, Building 12, 7491 Trondheim, Norway.; b Department of Psychology, The City College and Graduate Center of the City University of New York, New York City, United States of America.

Burnout has elicited growing interest among occupational health specialists in recent decades. Since 2019, the World Health Organization (WHO) has characterized burnout as a syndrome resulting from chronic, unmanageable workplace stress.^1^ According to the *International statistical classification of diseases and related health problems*, eleventh revision (ICD-11), three symptoms define the entity: (i) feelings of energy depletion or exhaustion; (ii) increased mental distance from one’s job or feelings of negativism or cynicism towards one’s job; and (iii) a sense of ineffectiveness and lack of accomplishment. The ICD-11 includes burnout among the factors influencing health status or contact with health services.

WHO’s definition of burnout closely corresponds to the definition inscribed in the Maslach Burnout Inventory, the most widely used measure of the entity.^2^^,^^3^ The Maslach Burnout Inventory approaches burnout as a syndrome induced by insurmountable work-related stress that comprises symptoms of exhaustion, cynicism and inefficacy.^2^ Exhaustion is considered burnout’s core. Released in 1981, the Maslach Burnout Inventory was the first standardized quantitative measure of burnout.^3^ The instrument consists of a questionnaire assessing the frequency of symptoms occurring over the past year. The Maslach Burnout Inventory played a key role in making burnout an object of investigation in occupational health science.

This perspective calls into question the definition of burnout embodied in the Maslach Burnout Inventory and incorporated into the ICD-11. We draw stakeholders’ attention to the fact that burnout’s symptoms and etiology were defined prior to any systematic research. We show that (i) exhaustion, cynicism and inefficacy do not form a cohesive syndrome; and (ii) no clear evidence exists that burnout is primarily caused by work-related stress. We discuss the implications of these findings for the status the ICD-11 grants to burnout.

## A predefined entity

A review of the early burnout literature reveals that the definition of burnout reflected in the Maslach Burnout Inventory was pre-established rather than derived from rigorous and replicable research. Maslach’s first paper on the issue, published in 1976, already described burnout in detail, at a time when no scientific study of burnout had been conducted. The author mentioned the fatigue, emotional overload, psychological distancing and withdrawal, cynical or negative attitudes, and sense of personal failing deemed to characterize affected individuals.^4^ The exhaustion, cynicism and inefficacy components of the Maslach Burnout Inventory were thus, in essence, all there. The paper even discussed variations in burnout rates, despite the absence of diagnostic criteria that might have allowed cases of burnout to be identified and counted.^4^

In addition to detailing the symptoms of burnout, this inaugural article approached the cause of the syndrome as if it were an elucidated issue. The author elaborated on the inability to cope with job stressors as the key etiological driver of burnout. Unresolvable job stress was presented as the factor to be acted upon to defeat burnout. The article, published in a social science magazine, took the form of a narrative report in which burnout was editorially treated as an established entity. No information was provided on the validity and reliability of the modus operandi that was followed to identify the symptoms and determinants of burnout.

Papers subsequently published by Maslach and her colleagues in the late 1970s capitalized on these prenotions and disseminated them further.^5^ These papers showed little anchorage in the literature on stress-related conditions available at the time. While several studies were publicized, their reporting was inadequate, making replication attempts challenging. Moreover, the reported studies were rudimentary in terms of design, measurement and data analysis. This body of work reaffirmed the authors’ preconceived views of burnout instead of subjecting those views to critical scrutiny and proper testing. The publication of the Maslach Burnout Inventory in the early 1980s crystallized burnout’s definition as a three-faceted syndrome induced by work-related stress.^3^ The questionnaire was cobbled together by factor-analysing a pool of items infused with the above-mentioned preconceptions. The Maslach Burnout Inventory was eventually copyrighted, which made its use chargeable. The release of the instrument legitimized the burnout construct, spurring research on burnout.

## An ill-defined entity

That burnout was largely predefined inevitably raises concerns about the sturdiness of the syndrome’s characterization.^5^^,^^6^ To this day, the reason for regarding exhaustion, cynicism and inefficacy as the signature symptoms of stressed-out workers remains unclear. Many authors have underlined that this symptom picture is clinically and theoretically ill-founded.^5^ In effect, the human response to unresolvable (job) stress involves a host of symptoms calling for serious examination, such as anhedonia, dysphoria, neurovegetative and psychomotor alterations, cognitive impairment or suicidality.^6^^,^^7^ The burnout construct overlooks most of these symptoms.

Researchers have further questioned the syndromal coherence of burnout.^6^ As a syndrome of exhaustion, cynicism and inefficacy, burnout is deemed distinct from long-identified stress-related conditions such as depression. Yet, exhaustion typically correlates more strongly with depressive symptoms than with cynicism and inefficacy.^6^ Such a pattern of results does not accord with the notion that burnout is a standalone syndrome. By definition, a syndrome refers to a set of co-occurring signs and symptoms. Because exhaustion co-occurs less frequently with cynicism and inefficacy than with depressive symptoms, it is unclear why cynicism and inefficacy are included in the syndrome when depressive symptoms are dismissed.

The idea that burnout primarily results from workplace stress is not better established. While numerous studies have documented links between job stressors and burnout, the most comprehensive meta-analysis available indicates that job stressors are weak predictors of burnout.^8^ Moreover, no conclusive evidence exists that workplace stress more specifically predicts burnout than it predicts, for example, depression.^9^ The paucity of research assessing job stressors with objective indicators further increases the uncertainty about the etiological link between job stressors and burnout. This body of findings is consistent with the limited effectiveness of organization-directed interventions for burnout.^10^ The view that work-related stress is the main cause of burnout lacks support.

Given the difficulty inherent in establishing credible causal links in psychological and psychiatric research, extensive and meticulous work is generally needed to produce causal inferences with any degree of confidence. Surprisingly, the pioneers of burnout research drew immediate conclusions regarding burnout’s etiology. However, the predictors of burnout are not the only source of concern. The sequelae of burnout are also open to question. For instance, although burnout is expected to severely undermine an individual’s ability to work, only tenuous links between burnout and objective job performance have been documented.^11^ Both ends of the burnout chain thus appear to require reconsideration.

## An elusive syndrome

The confusion surrounding burnout’s definition is further discernible on a diagnostic level. Despite nearly 50 years of research, no valid diagnosis for the syndrome exists.^6^^,^^12^ The inability to generate and validate clear diagnostic criteria for burnout raises additional doubts about the construct’s content. If exhaustion, cynicism and inefficacy formed a well-defined, cohesive syndrome, affected individuals should be identifiable. Clinicians have pointed out that burnout may simply be too loose and artificial an entity for a diagnosis to be workable.

Despite the absence of a valid diagnosis, burnout is often portrayed as rampant. The burnout epidemic narrative is based on studies that estimated burnout prevalence with arbitrary and elastic criteria.^12^ The use of lenient categorization criteria has been particularly problematic. Such criteria pathologize everyday dissatisfaction and discomfort instead of targeting individuals who truly need assistance. As a result, interventional resources are likely to be misdirected, and their impact diluted.

Due to its extensive use, the burnout label may now commonly mask depressive conditions, increasing the risk of depression going underdiagnosed and untreated.^6^^,^^9^ This state of affairs is concerning. Depression is associated with enormous health, social and economic costs, and can lead to suicide. The diverting effect of the burnout label thus poses a multifaceted and ethical problem.

## Conclusion

Disconcerting as it may be, no clear evidence has emerged for the existence of a work-induced syndrome of exhaustion, cynicism and inefficacy, leaving burnout as a catchy but confusing label.

Multiple alternate definitions of burnout have been produced over the years. Unfortunately, these alternate definitions have inherited fundamental flaws from Maslach’s preconception of burnout, such as a questionable symptom scope, a lack of clinical underpinning and an overlap with existing conditions.^6^^,^^13^ Interestingly, burnout has sometimes been equated with neurasthenia, another condition marketed as a malady of modern civilization.^5^^,^^14^ Neurasthenia was removed from the ICD-11 because of its vagueness and lack of clinical validity.

We call for a revision of burnout status, leveraging WHO’s evidence-based procedures for consensus-building. The various stress, anxiety and depressive disorder categories available in the ICD-11 offer plenty of solutions for addressing job-related distress. However, a recommendation to investigate work-related adversity during diagnostic processes could be formally included if deemed useful. Alternatively, given the overlap of burnout with depression,^6^^,^^9^ an occupational depression^13^ qualifier could be added to the depressive disorder category. Whatever solution is preferred, we suggest deleting the burnout category from the ICD-11. 
